# Indicator Selection for Life Prediction of Polyimide Enameled Wire for Aviation Generators and Method for Establishing Life Curve—Based on Bayesian Nonlinear Regression

**DOI:** 10.3390/polym18111343

**Published:** 2026-05-28

**Authors:** Zihan Wang, Yongzhi Liu, Tianxing Li, Peirong Zhu, Guodong Niu, Haoran Du

**Affiliations:** Air Force Engineering University, Xi’an 710038, China; 18295028691@163.com (Z.W.); litianxing2011@163.com (T.L.); r1933968982@163.com (P.Z.); 17885079664@163.com (G.N.); duhaoran19991008@163.com (H.D.)

**Keywords:** polyimide, winding, accelerated thermal aging, lifetime prediction, aviation generator

## Abstract

Insulation failure in aviation generator windings is one of the most common faults. Modern aircraft winding materials often employ polyimide enameled wire, making research on its reliability and health monitoring particularly important. Based on the relationship between temperature and aging rate described by the Arrhenius law, this study designed accelerated thermal aging experiments, testing twisted-pair, coil, and winding samples made of copper-core polyimide enameled wire. The variation in multiple parameters was visualized using B-spline fitting, ultimately identifying parallel equivalent capacitance as the most suitable parameter for monitoring generator winding insulation. It was also indicated that aging of the winding insulation coating has almost no effect on the performance of the electrical system. Finally, experimental data were processed using Bayesian nonlinear regression, where prior data were updated with new data to obtain posterior aging curves. When the IC ($C_{p}$) value reaches 1.2009 and 1.4089 times its initial value, the sample is considered to have reached 50% and 100% of its lifespan, respectively. This provides a reference approach and quantitative indicators for predicting the lifespan of polyimide enameled wire windings.

## 1. Introduction

Aviation generators are the core of the aircraft power system. Their primary function is to convert the kinetic energy from the engine into electrical energy and supply power to all electrical equipment onboard, making them critical components in modern aircraft. They operate under relatively harsh conditions, and any failure may lead to serious consequences for flight safety [1,2]. Among common generator failures, insulation failure in the windings is one of the most frequent [3,4]. As modern aircraft predominantly employ polyimide enameled wires for windings, research on their reliability and health monitoring is particularly important.

Polyimide (PI) is a class of high-performance polymer materials containing cyclic imide groups in the molecular chain. It is synthesized through polycondensation, film casting, and imidization processes from pyromellitic dianhydride and diaminodiphenyl ether, resulting in a high-performance insulating material. It exhibits a broad operating temperature range and high mechanical strength. When used as an insulating coating for enameled wires, it maintains stable dielectric properties, making it widely applicable in slot insulation and enameled wires for aviation motors. However, the operating environment of aviation generator windings is often harsh, subject to multi-stress coupling aging effects including thermal, electrical, mechanical, and environmental stresses, among which thermal stress is the primary aging factor [5,6,7]. For the aging monitoring of enameled wires, the most widely accepted indicators include IC (insulation capacitance), tan δ (dielectric dissipation factor), PDIV (partial discharge inception voltage), among others. In previous studies, researchers primarily fabricated corresponding samples and conducted offline aging experiments focused on the material’s characteristics [7,8]. Literature [4] also points out that IC is an effective parameter for characterizing the aging level of enameled wires. Reference [9] points out that for aviation generator windings, L and R are also important parameters affecting their operating state, and in modeling, the windings are often regarded as a resistance-inductance element. However, research in related fields still has certain limitations. Reference [10] only studied twisted-pair wire samples, with a relatively single type of specimen. In the study of Reference [11], although multiple specimens such as twisted-pair wires, motorettes, and coils were investigated, the connection with actual motor applications was insufficient. In the studies of References [12,13], although actual motors and winding products were examined, their monitoring methods require additional installation of current probes or signal generators, making them suitable for ground-based systems but not feasible for implementation on aircraft.

Polyimide, as a commonly used insulating material for enameled wires, has a relatively wide range of applications. However, research on its aging characteristics remains insufficient. Due to its high-temperature resistance and stable chemical and electrical properties [14], accelerated aging experiments on polyimide enameled wires are characterized by long cycles, high material consumption, and significant experimental costs [15]. Consequently, publicly available datasets are scarce, and obtaining aging data is challenging. Further research is needed to extend the understanding of its properties from the material itself to finished winding products and to develop health monitoring methods. The objective of this study is to conduct accelerated thermal aging experiments on polyimide enameled wire samples and finished winding products based on the Arrhenius law, to identify a suitable parameter for characterizing aging, and to use the B-spline interpolation method to obtain the relationship curve between this parameter and insulation lifespan. The curve derived from finished windings will be used to adjust the curve obtained from enameled wire samples through Bayesian learning, ultimately yielding a curve that accurately reflects the lifespan of polyimide enameled wires in aviation generators. This will provide a basis for lifespan prediction and health monitoring of aviation generators.

## 2. Materials and Methods

### 2.1. Sample Preparation

This experiment selected a commonly used enameled wire model for aviation generators, with the following detailed technical parameters:

- Standard: IEC 600317-0-1:2005 [16]
- Temperature Class: 220 °C
- Conductor Diameter: 1.0 mm
- Conductor Material: Round copper wire
- Insulation Coating Material: Polyimide
- Insulating Film Thickness: Grade 2 (minimum film thickness 0.016 mm)

**Figure 1:**
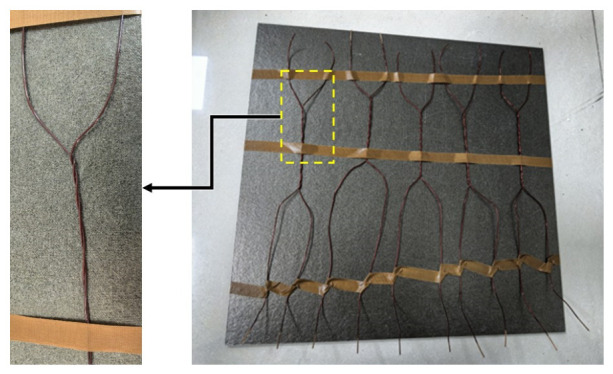
A group of completed enameled wire samples.

To investigate the aging characteristics of the polyimide material itself and to establish aging curves for the classic aging characterization parameters IC and tan δ, this study fabricated twisted-pair samples in accordance with standard [IEC 60851-5:2019] [17]. These samples were grouped with five samples per set, secured onto mica boards using PTFE tape, as shown in Figure 1.

Since both PTFE tape and mica boards exhibit excellent high-temperature resistance, the twisted-pair samples fixed with them can maintain a stable physical state, ensuring that any parameter changes are solely due to the aging of the insulating material [10].

**Figure 2:**
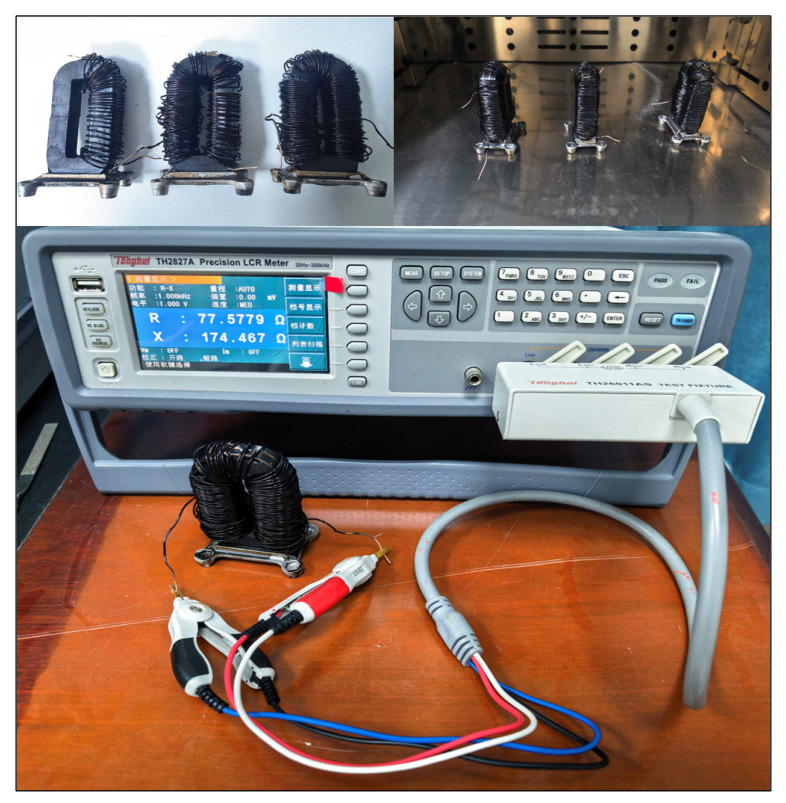
Polyimide enameled wire coil samples with iron cores and their thermal aging experiment.

Considering that in practical applications, the L and R of the windings are important indicators of their electrical characteristics, and their changes can significantly impact the entire power system while being easily monitored, L and R should also be included as reference parameters in the health monitoring of windings. To investigate the effect of insulation aging on L and R, this study constructed coil samples with an iron core using enameled wire of the same specifications as those of the twisted-pair wire samples. The iron core was made of silicon steel sheets, as shown in Figure 2.

The specimens shown in Figure 2 were fabricated by winding the aforementioned enameled wire—with lengths of 3 m, 15 m, and 20 m, respectively—in the same winding direction around an iron core. This set of coil specimens simulates windings with different numbers of turns. This experiment was conducted in two rounds, involving a total of six samples.

**Figure 3:**
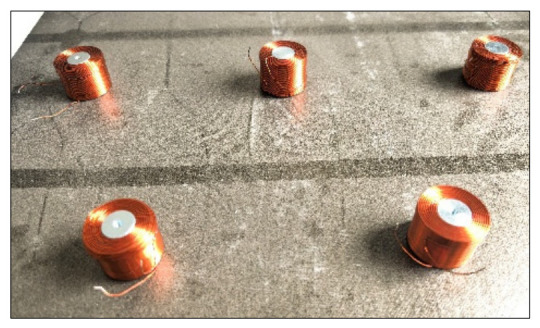
Finished winding specimens for accelerated aging experiment.

Due to the difficulty in obtaining genuine aviation generator windings and their high cost, it is challenging to conduct accelerated aging experiments using a large number of such samples. In light of these constraints, this study also adopted a research method suitable for small sample sizes, performing accelerated aging experiments on five finished winding specimens as depicted in Figure 3.

### 2.2. Accelerated Aging Experimental Design

The Arrhenius equation reveals the quantitative relationship between the rate constant of a chemical reaction and temperature and is one of the most commonly used theories for evaluating the thermal aging lifespan of insulating materials [18]. Its formula is as follows:

(1) $$k=Ae^{-\frac{E_{a}}{RT}},$$

where k is the reaction rate constant, which determines the rate of the reaction; A is the pre-exponential factor, which is related to the frequency of molecular collisions; $E_{a}$ is the activation energy (unit: “J/mol”), that is, the energy barrier that must be overcome for the reaction to occur. The higher the activation energy, the better the thermal stability of the material; R is the molar gas constant, with a value of 8.314 “J/(mol·K)”; T is the absolute temperature.

In the fitting of thermal aging experimental data, the equation is usually transformed by taking the logarithm of both sides [19], converting it into the following linear form:

(2) $$ln\left(L\left(T\right)\right)=\ln\left(L_{0}\right)+\frac{E_{a}}{k}\cdot \frac{1}{T},$$

where $ln\left(L\left(T\right)\right)$ is the logarithm of thermal life; $\frac{1}{T}$ is the reciprocal of absolute temperature; $\ln\left(L_{0}\right)$ is the intercept; $\frac{E_{a}}{k}$ is the slope.

The Arrhenius law reveals that the effect of temperature on life is exponential. Based on this, Monsinger’s law (i.e., the ten-degree rule) also states that in the thermal aging study of polymer materials, for every 10 °C increase in temperature, the chemical reaction rate approximately doubles [20]. In the accelerated thermal aging experiment of twisted-pair samples, the temperature interval is usually 20 °C [21]. Therefore, for the polyimide enameled wire with a temperature class of 220 °C, this study selected three temperature environments: 310 °C, 330 °C, and 350 °C, using a high-temperature oven (senyu DHG500-2, Shenzhen, China, power supply: 380 V three-phase AC) for constant-temperature accelerated aging. For the accelerated thermal aging experiment of coil samples and finished windings, to accelerate the experimental process, 350 °C was selected as the temperature for the accelerated aging experiment, and the appropriate number of aging cycles should be chosen as the life termination condition based on the experimental results of the twisted pair samples. Before conducting the aging experiment, the samples need to be wiped with anhydrous ethanol to remove surface impurities. The detailed experimental steps are as follows:

1. Set the oven temperature to the experimental design value and set the time to 4 h;
2. After the oven completes preheating and the temperature reaches the set value, place the samples into the oven;
3. The samples undergo constant-temperature accelerated thermal aging. After the set time (4 h) is reached, the oven alarm sounds;
4. Remove the samples and allow them to cool naturally to room temperature;
5. Determine whether they have reached the end-of-life criterion;
6. If the samples have not reached the end of life, measure their corresponding parameters;
7. Record the test results and continue the next round of aging tests for the samples that have not reached the end of life.

The end-of-life criterion for the twisted-pair samples was the withstand voltage test, and its experimental procedure has been described in detail in previous research [22]. It should be noted that this paper has adopted the accelerated aging experimental method used in the previous study and employed the same experimental equipment. Similar methods are widely applied in the industry. The research in this paper builds upon the foundation of the previous study and makes further expansions and innovations. It has updated a large number of experiments and achieved further breakthroughs and results.

### 2.3. Measurement of Relevant Dielectric Parameters

The testing conducted on the twisted-pair wire samples of polyimide enameled wire represents a classic thermal endurance evaluation aimed at investigating the intrinsic material properties of this type of enameled wire. Therefore, two conventional aging parameters IC and tan δ, were selected.

In contrast, the tests on the polyimide coil samples with an iron core were primarily designed to examine whether the three parameters—L, R and $C_{p}$—which critically determine the electrical performance of the enameled wire, exhibit clear and regular variations as the enameled wire ages. Additionally, the tests aimed to explore whether the aging of the enameled wire would significantly impact the overall electrical performance of the system.

The measurement of $C_{p}$ was intended to refine the aging curves based on experimental results from the twisted-pair wire samples, thereby enabling the derivation of final conclusions and the plotting of aging characteristic curves. $C_{p}$ refers to the parallel equivalent capacitance of the winding, which is collectively determined by factors such as inter-turn capacitance ($C_{i}$) and turn-to-core capacitance ($C_{m}$). As indicated in reference [12], $C_{i}$ dominates $C_{p}$ by orders of magnitude compared to other influencing factors, and thus $C_{p}$ can be considered primarily determined by $C_{i}$.

Finally, for the testing of the finished polyimide windings specimens, two parameters—L and R—were selected for measurement. The primary purpose of measuring L and R was to cross-verify the experimental results obtained from the coil samples.

All tests were conducted at room temperature and under atmospheric conditions. The power supply for all testing instruments was 220 V AC.

#### 2.3.1. Measurement of IC and tan δ for Polyimide Twisted-Pair Wire Samples

**Figure 4:**
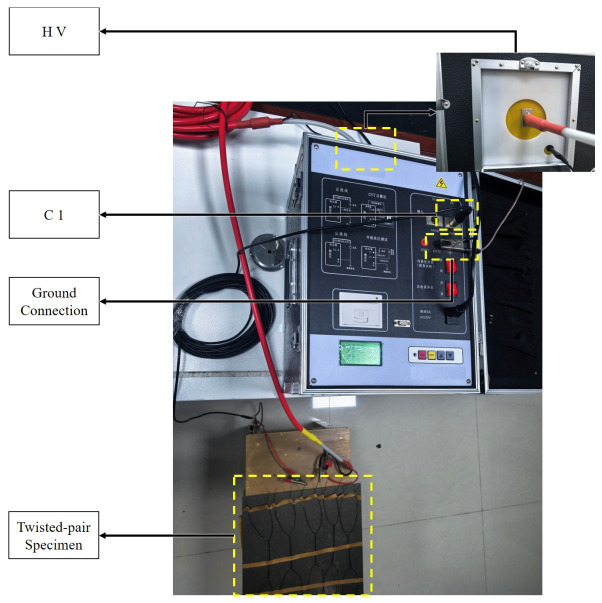
Dielectric dissipation tester and its connection method. Adapted from [22], electronics, 2026.

The measurement of IC and tan δ for the twisted-pair samples was performed using a dielectric dissipation tester (Xike XKJS-E, Yangzhou, China). The specific wiring method and physical diagram are shown in Figure 4.

The experiment employed the direct connection method, with a test frequency of 50 Hz and a test voltage of 0.5 kV. Upon initiating the test, the instrument emitted an audible prompt, which continued until the calculation results were obtained, after which the prompt ceased, and the data was recorded.

**Figure 5:**
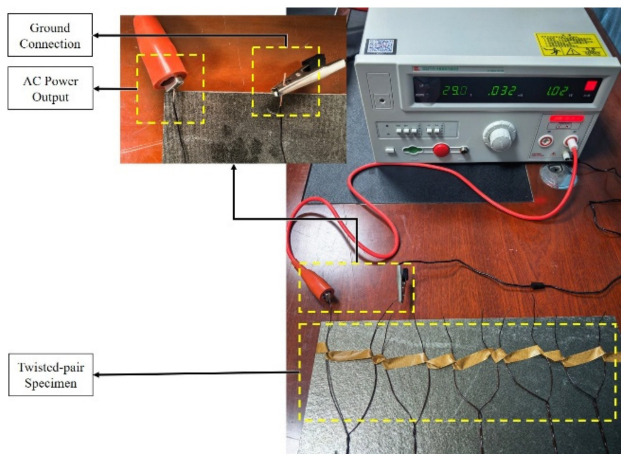
Withstand voltage tester and its connection method. Adapted from [22], electronics, 2026.

Determining the end of life required a withstand voltage test conducted using a withstand voltage tester (CHANGSHENG CS2671BX, Nanjing, China), as shown in Figure 5.

The test voltage was set to 500 ${V\;}_{rms}$ with a frequency of 50 Hz, and the test duration was set to 60 s. If the sample was not broken down, it was considered to have passed the withstand voltage test and would proceed to the next cycle of aging; otherwise, the tester will issue an audible and visual alarm, indicating failure to pass the withstand voltage test and that it is deemed to have reached its end-of-life criterion.

#### 2.3.2. Measurement of L, R and $C_{p}$ for Polyimide Coil Samples

The L and R of the coil samples were measured using a Precision LCR Meter (Tonghui TH2827A, Changzhou, China). During testing, both ends of the coil were connected to the two test clips of the instrument, as shown in Figure 2. The test frequency was set to 1.0 kHz. For measuring L, the instrument mode was adjusted to $L_{s}$-Q; for measuring R, the mode was adjusted to R-X. When measuring the $C_{p}$, the wiring method remained the same, but the test frequency needed to be adjusted to 120 Hz and the mode to $C_{p}$-D.

#### 2.3.3. Measurement of L and R for Polyimide Winding Specimens

The measurement of L and R for the finished winding specimens was similar to that for the coil samples. The purpose of establishing this experimental group was to eliminate uncertainties introduced by manual fabrication of the coil samples and to cross-validate the experimental conclusions with those derived from the coil samples tests.

## 3. Results

**Figure 6:**
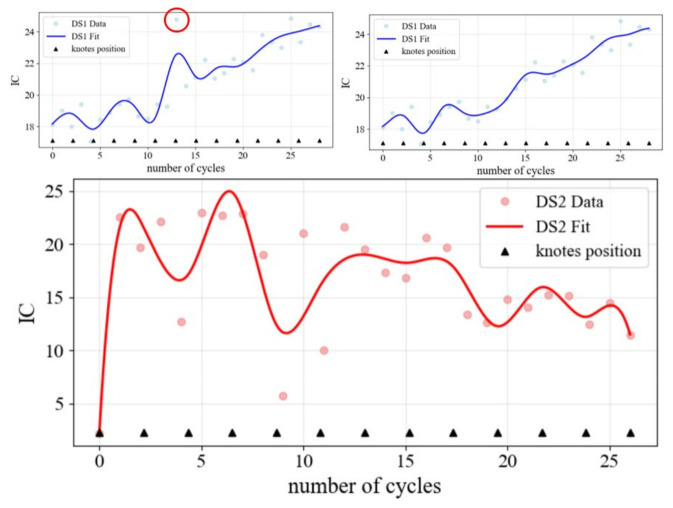
Graph lines for data preprocessing (The area within the red circle indicates abnormal data).

This chapter is structured in three parts, which, respectively, present the experimental data for three types of samples and provide preliminary processing of these data. After obtaining the experimental data, preprocessing is required, as illustrated in Figure 6.

In Figure 6, for data points that deviate significantly from the overall trend, this study replaces them with the average value of the two adjacent data points. For curves that exhibit a trend distinctly different from the other samples in the same group and show abnormal fluctuations, they are considered potentially erroneous due to reasons such as inherent defects in the individual sample or inadvertent experimental operation. Consequently, the entire dataset from that group is deemed non-representative and is discarded in its entirety.

The fitting of experimental data was performed using the B-spline interpolation algorithm. B-spline curves enable data interpolation with controlled continuity and smoothness while effectively preserving local geometric features [23,24]. The fundamental concept is to connect multiple low-degree polynomial curve segments and construct a smooth curve guided by a set of control points. These control points form a control polygon that defines the global shape of the curve. A knot vector, which is a non-decreasing sequence of real numbers, governs the properties of the B-spline basis functions, thereby regulating the influence range of each control point on the final curve.

In B-spline fitting, the degree *p* is typically set to 3 (i.e., cubic B-spline), defining the polynomial degree of each curve segment. The B-spline basis function is formally defined as follows:

Zero-degree basis function (p = 0):

(3) $$N_{i,0}(u)\left\{\begin{array}{c} 1\;\;if\;u_{i}\leq u\leq u_{i+1}\\ 0\;\;\;\;\;\;\;\;\;\;\;\;\;\;else\;\;\;\;\;\;\;\;\;\;\; \end{array}\right.,$$

Higher-degree basis functions (p > 0):

(4) $$N_{i,0}\left(u\right)=\frac{u-u_{i}}{u_{i+p}-u_{i}}N_{i,p-1}\left(u\right)+\frac{u_{i+p+1}-u}{u_{i+p+1}-u_{i+1}}N_{i+1,p-1}\left(u\right),$$

This function demonstrates that a B-spline basis function of degree p is constructed through a linear combination of two basis functions of degree (p−1), ensuring the smoothness of the resulting curve.

To quantitatively evaluate the risk of overfitting, this study employs K-fold cross-validation (with K=5) to determine the appropriate number of knots. K-fold cross-validation is a robust method for assessing model generalization performance. It involves partitioning the training data into K subsets, iteratively using K−1 subsets for model training and the remaining subset for validation, and finally averaging the results from the K iterations to obtain a final performance metric. This approach mitigates the random fluctuations that may arise from a single, arbitrary partition of the data.

Finally, based on the above validation results, this study presents B-spline interpolation curves and residual distribution plots, which, respectively, illustrate the preliminary processed data and the quality assessment of the curve fitting.

### 3.1. Thermal Aging Experimental Data of Polyimide Twisted-Pair Samples

**Figure 7:**
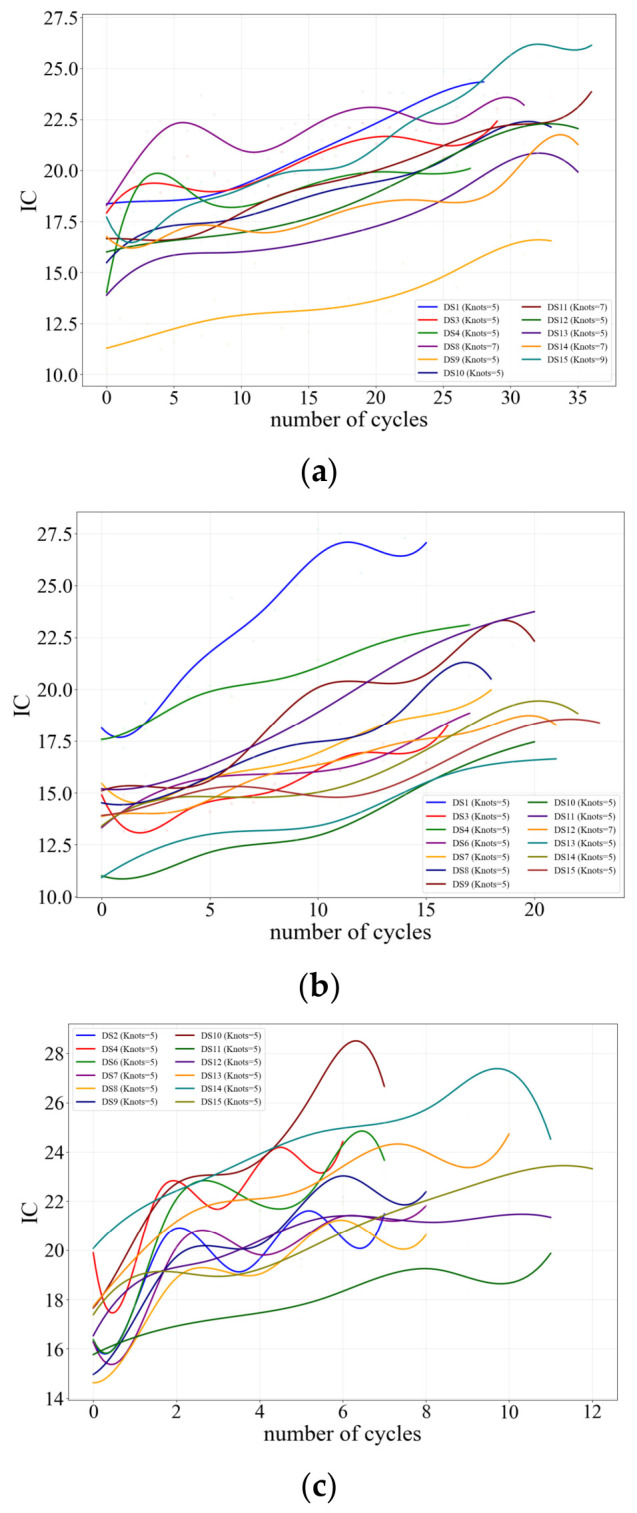
Variation curves of IC values in accelerated thermal aging experiments at 310 °C (**a**), 330 °C (**b**), and 350 °C (**c**). Adapted from [22], electronics, 2026.

As shown in Figure 7, the IC (unit: pF) exhibits a monotonically increasing trend during the accelerated thermal aging experiments conducted at 310 °C, 330 °C, and 350 °C.

In Figure 7, the IC values of each group of samples increase with the progression of aging. Although they have different initial values, the trend and magnitude of their changes are basically the same. Due to unavoidable experimental errors, the curves exhibit some fluctuations, but overall, they are consistent with theory. When the sample life ends, IC reaches the maximum value.

**Figure 8:**
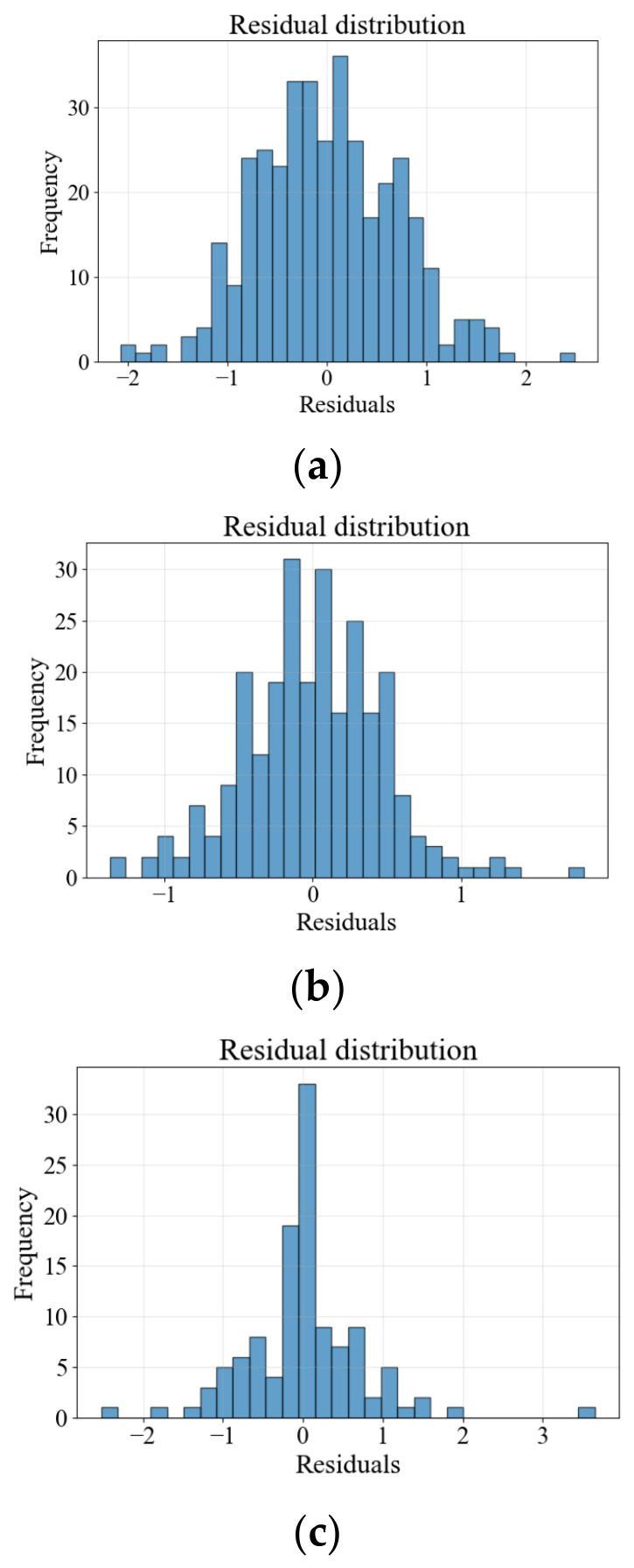
Residual plot of fitted IC values from accelerated thermal aging experiments at 310 °C (**a**), 330 °C (**b**), and 350 °C (**c**). Adapted from [22], electronics, 2026.

The residual distribution plot for the corresponding data is as follows:

In Figure 8, the residual values for all three sets of data approximately follow a normal distribution, indicating a good fitting performance.

**Figure 9:**
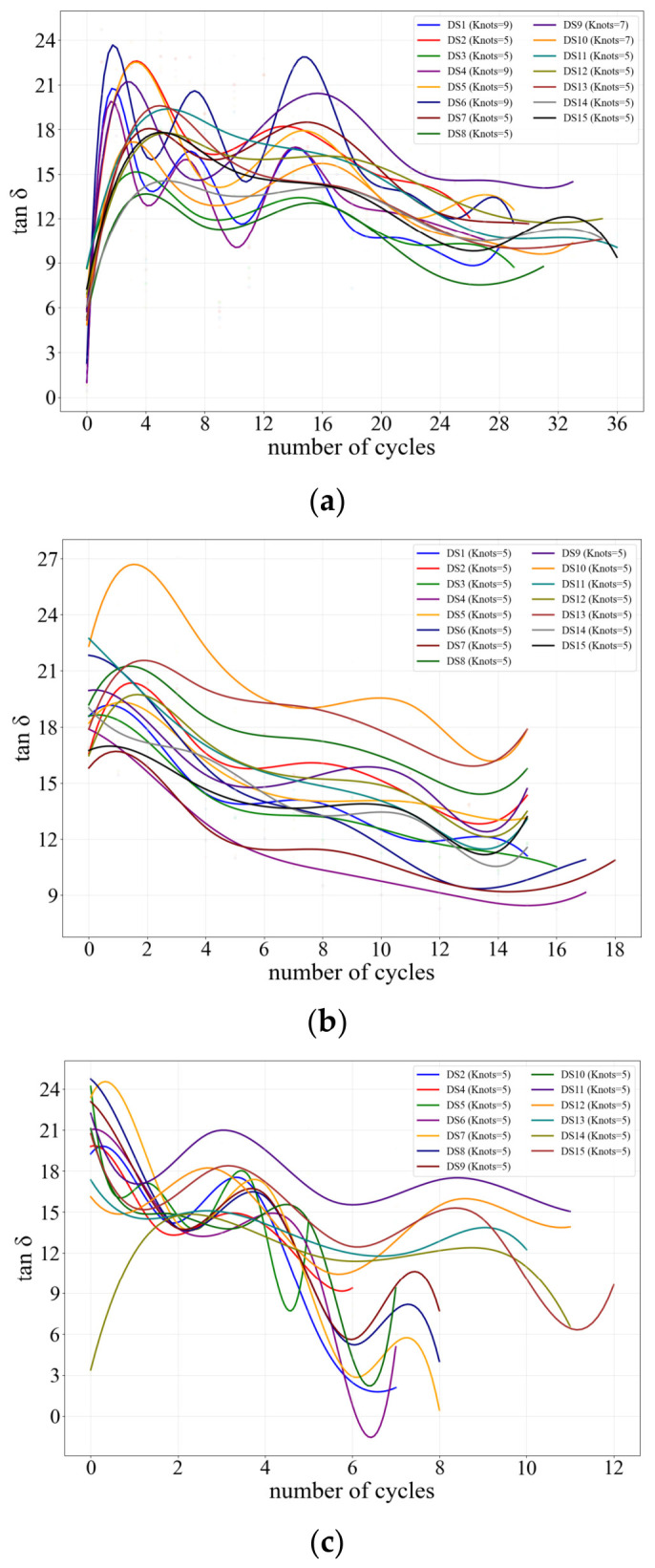
Variation curves of tan δ values in accelerated thermal aging experiments at 310 °C (**a**), 330 °C (**b**), and 350 °C (**c**). Adapted from [22], electronics, 2026.

Unlike the IC, the variation of tan δ does not exhibit clear monotonicity and therefore lacks reference value, as shown in Figure 9.

In Figure 9, it can be clearly observed that the tan δ values exhibit significant fluctuations and fail to demonstrate the expected increase with the progression of aging, making it difficult to reflect a monotonic increasing pattern related to thermal lifespan.

Analysis suggests that this may be caused by variations in environmental factors, such as air humidity, between different measurement sessions. Literature [21] also indicates that tan δ is highly susceptible to environmental influences, which could lead to less ideal measured data. Thus, it further demonstrates that tan δ may not be suitable as an aging indicator under certain conditions. It should be noted that the experimental data in this section are derived from my previous research [22], and in this study, I have reorganized, analyzed and plotted them. The data in the next two sections are obtained from further research.

### 3.2. Thermal Aging Experimental Data of Polyimide Coil Samples

**Figure 10:**
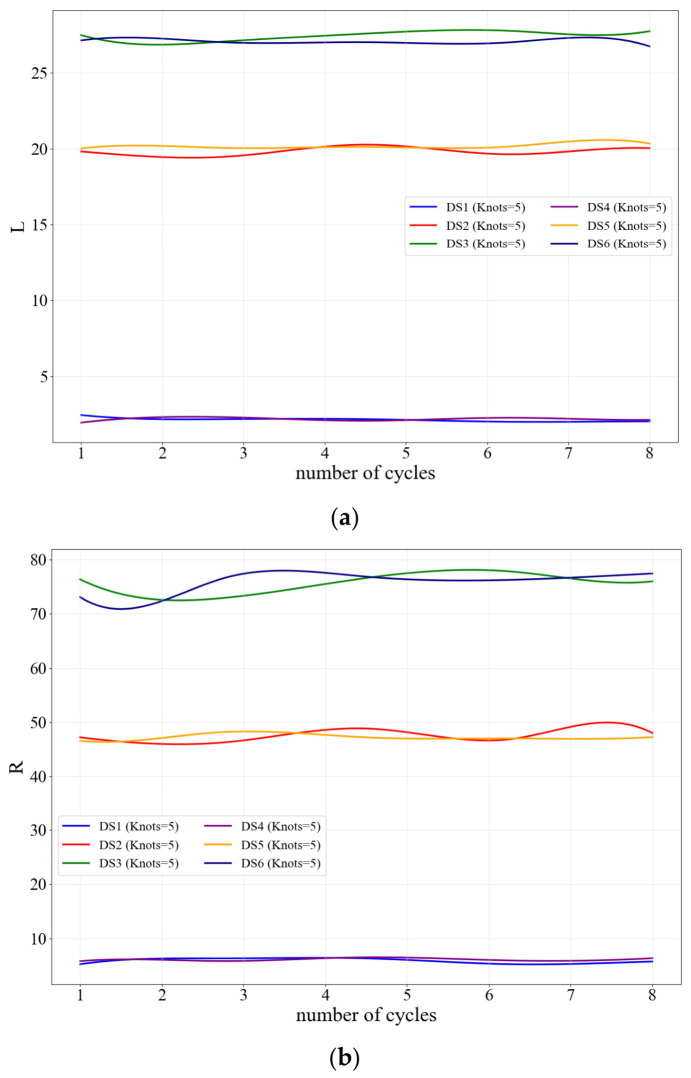

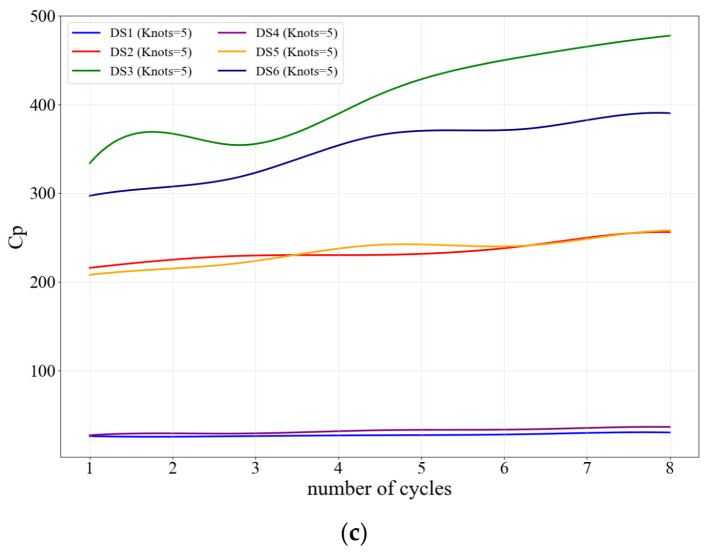
Variation curves of L (**a**), R (**b**) and $C_{p}$ (**c**) values from the accelerated thermal aging experiment of coil samples.

Based on the accelerated aging tests of twisted-pair samples, it was found that their aging behavior follows the Arrhenius law. After nine cycles of accelerated aging at 350 °C, more than half of the samples had reached their end of life. Ref. [11] also indicates that for enameled wire samples of the same specification but with different winding methods, the same test standards can be applied. Furthermore, refs. [25,26] demonstrate that the thermal aging mechanism of PI materials does not change under conditions of 350 °C, indicating no risk of mechanism deviation. Therefore, to achieve the fastest possible test progress while referring to the conclusions of completed experiments, the accelerated aging temperature for this test was set at 350 °C. Since the enameled wires used for preparing various sample types are of identical specifications, this test will adopt the conclusion drawn from the twisted-pair samples and set the aging cycle number for coil samples at eight. Exceeding eight cycles (32 h) is considered to have reached the end-of-life condition, with the corresponding changes in L and R data shown in Figure 10.

Figure 10 indicates that the enameled wire exhibits minimal changes in L (unit: mH) and R (unit: Ω) values with aging, with no consistent pattern observed. Given the unavoidable experimental errors, it can even be considered that these parameters remain essentially unchanged. Therefore, it can be concluded that the aging of the polyimide insulation coating on the enameled wire does not significantly affect the electrical performance of the system. The trend of $C_{p}$ (unit: μF) change is essentially the same as that of IC, which aligns with the experimental expectations.

### 3.3. Thermal Aging Experimental Data of Polyimide Finished Winding Specimens

**Figure 11:**
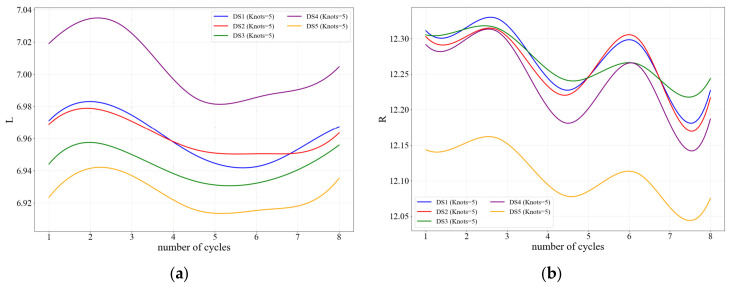
Variation curves of L (**a**) and R (**b**) values from the accelerated thermal aging experiment of finished winding specimens.

The end-of-life criterion for the finished winding specimens is the same as that for the coil samples, both determined to be 8 cycles based on the experimental results of the twisted pair samples. The corresponding changes in L and R are shown in Figure 11.

Figure 11 shows that the measured results for L (unit: mH) and R (unit: Ω) are similar to those of the previous coil samples, providing mutual corroboration and supporting the earlier conclusions.

## 4. Discussion

### 4.1. Arrhenius Curve

Based on the experimental data of twisted-pair samples at different temperatures, this study obtained the mean thermal life values at 310 °C, 330 °C, and 350 °C, as shown in the table below.

**Figure 12:**
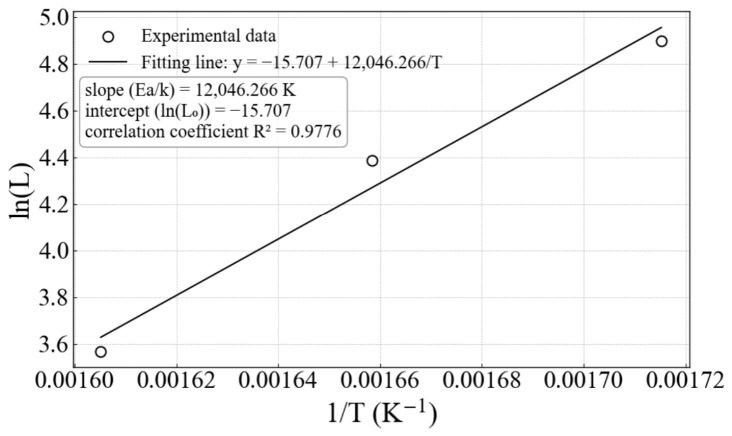
The Arrhenius curve derived from data fitting. Adapted from [22], electronics, 2026.

The data from Table 1 were fitted using the Arrhenius equation, and the results are shown in Figure 12.

Figure 12 indicates that the thermal aging characteristics of the polyimide material generally follow the Arrhenius law. The relationship between thermal life and aging temperature can be determined using the correlation shown in the figure. In subsequent studies, depending on the required number of aging cycles for the experiment, the appropriate temperature for accelerated thermal aging experiments can be determined based on the above relationship.

### 4.2. Normalization Processing

**Figure 13:**
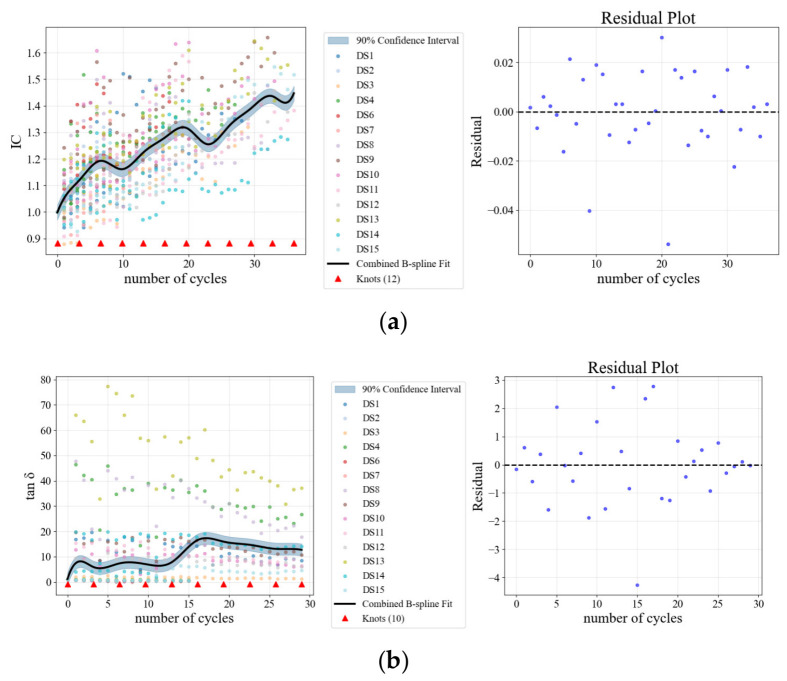
Curves of IC (**a**) ($R^{2}$ = 0.9791) and tan δ (**b**) ($R^{2}$ = 0.8983) values for twisted-pair samples after initial value normalization.

Due to the inevitable inherent individual differences among various samples, the initial values of their respective parameters are not identical. To enhance generalizability and establish a unified standard, this study normalizes the initial values of each dataset, measuring the degree of change as a percentage relative to the initial parameter values. The data after initial value normalization are as follows:

Figure 13 presents a fitted curve derived from the pooled dataset of twisted-pair samples after initial value normalization at three temperatures. This curve reflects the properties of the polyimide material. The figure also reveals that when the IC value increases by approximately 20%, the twisted-pair samples begin to exhibit potential failure. When the IC value increases by approximately 60%, all twisted-pair samples have experienced insulation failure. However, after initial value normalization and fitting into a single curve, the tan δ value still does not serve as a reliable indicator and is not suitable for characterizing the thermal life of the enameled wires.

**Figure 14:**
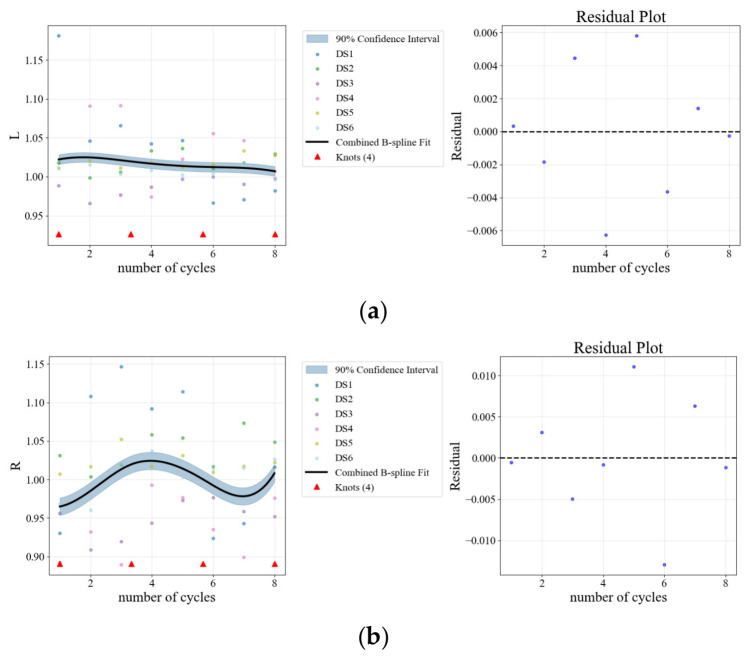

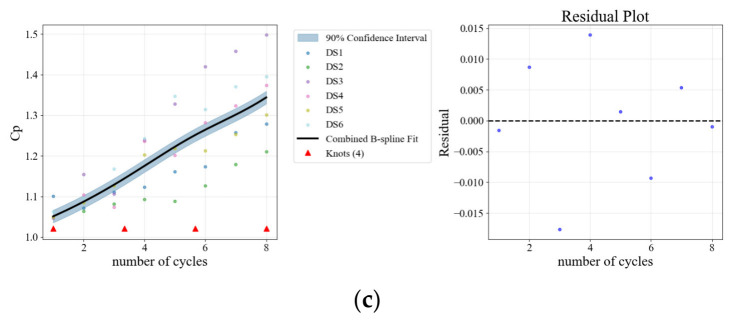
Curves of L (**a**) ($R^{2}$ = 0.7020), R (**b**) ($R^{2}$ = 0.8898) and $C_{p}$ (**c**) ($R^{2}$ = 0.9908) values for coil samples after initial value normalization.

Figure 14 shows the curves of the L and R values of the coil samples after initial value normalization. The figure indicates that after the dataset is pooled and fitted to a single curve, the L and R parameters of the samples exhibit minimal fluctuations, with the curve trending toward greater stability. Furthermore, the fluctuations show no discernible pattern. The variation in the $C_{p}$ value, however, shows a trend similar to that of the IC value of the twisted-pair samples, demonstrating an increasing trend with aging time.

**Figure 15:**
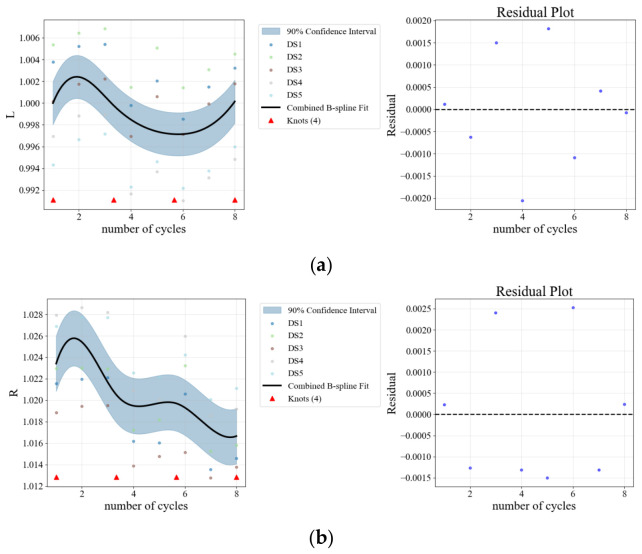
Curves of L (**a**) ($R^{2}$ = 0.6701) and R (**b**) ($R^{2}$ = 0.7586) values for finished winding specimens after initial value normalization.

Figure 15 displays the curves of the L and R values for the finished windings. The L and R values exhibit characteristics similar to those of the coil samples, enabling cross-verification with them.

### 4.3. Bayesian Curve

Bayesian learning is a statistical learning method based on Bayes’ theorem. It treats model parameters as random variables and updates the understanding of their uncertainty through observed data. Its core idea is to describe all unknown quantities (parameters, models, predictions) in the language of probability. After acquiring data, it combines prior knowledge with evidence from the data to obtain the posterior distribution, upon which inference and prediction are then based.

Bayes’ theorem is given by:

(5) $$p\left(\theta |D\right)=\frac{p\left(D|\theta \right)\;p\left(\theta \right)}{p\left(D\right)}\propto p\left(D|\theta \right)\;p\left(\theta \right),$$

where $p\left(\theta \right)$ is the prior distribution, representing the initial belief about the parameters before being updated by new data; $p\left(D|\theta \right)$ is the likelihood function, indicating the probability of observing the data given the parameters; p(D)=∫p(D∣θ)p(θ) dθ is the marginal likelihood, which serves as a normalization constant and usually does not need to be explicitly computed; and $p\left(\theta |D\right)$ is the posterior distribution, which represents the updated probability distribution of the parameters after incorporating the data and serves as the core output of Bayesian learning [27].

Bayesian nonlinear regression is a statistical learning method that integrates Bayesian inference with nonlinear models. It is capable of capturing complex nonlinear relationships in data while naturally quantifying uncertainties in both parameter estimates and predictions. Rather than merely providing point predictions, the method yields predictive distributions along with credible intervals, which are critical for decision-making and risk-sensitive applications. By incorporating prior knowledge, Bayesian nonlinear regression produces results that represent a balance between prior information and observed data. This characteristic makes it particularly suitable for small-sample scenarios, such as those often encountered in materials modeling [28]. In this study, the IC data of the twisted pairs are used as the prior distribution, and the $C_{p}$ data of the finished windings are treated as the new data. Through Bayesian nonlinear regression, the new data are used to update the prior distribution, resulting in the posterior distribution, which serves as the final lifetime curve for the thermal aging of the windings.

**Figure 16:**
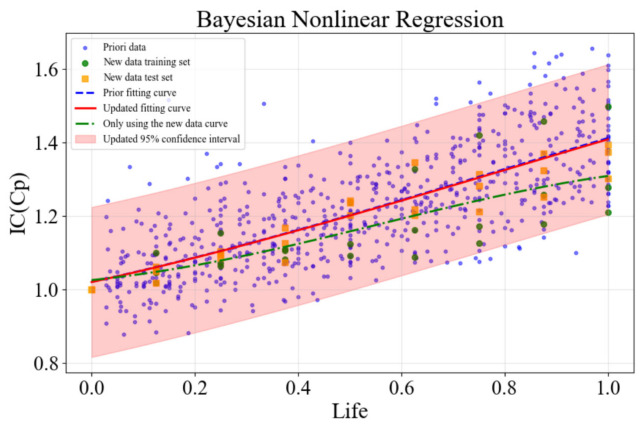
Corrected $C_{p}$ posterior distribution.

Using Bayesian nonlinear regression offers distinct advantages over traditional methods. Firstly, winding data experiments are costly and difficult to obtain, often resulting in small sample sizes that are prone to overfitting. However, in Bayesian nonlinear regression, the prior distribution imposes constraints on the new data, effectively mitigating overfitting. Secondly, Bayesian nonlinear regression possesses an adaptive weighting capability, which allows it to appropriately determine the weights of new and existing data without requiring manual setting. Lastly, Bayesian nonlinear regression exhibits strong robustness: it automatically widens the confidence intervals, providing a degree of tolerance for outliers in the data. The corrected posterior distribution curve is as follows:

Figure 16 presents the aging curve, i.e., the lifetime function, obtained through Bayesian nonlinear regression. This curve reflects the relationship between the IC (C_p_) value and the insulation lifetime of the enameled wire. The horizontal axis represents the entire lifespan of the sample, with 0.0 indicating the beginning of life and 1.0 indicating the end of life. The vertical axis represents the normalized IC (C_p_) value of the sample, where 1.0 corresponds to the initial value of the sample. The blue curve represents the prior data, derived from the twisted-pair sample data; the green curve is the fit obtained using only the new data, i.e., the coil sample data; and the red curve represents the posterior distribution curve, which updates the prior distribution with the new data. Since the prior distribution and the new data exhibit essentially the same trend, the posterior distribution also shows a monotonically increasing trend of the C_p_ value with the progression of aging. The shaded area around the curves indicates the 95% confidence interval. From the curve, it can be observed that the IC (C_p_) value increases monotonically with the aging of the sample. When the IC (C_p_) value reaches 1.2009 times its initial value, the insulation lifetime of the sample reaches 50%; and when the IC (C_p_) value reaches 1.4089 times its initial value, the sample’s lifetime ends.

### 4.4. Experimental Conclusion Analysis and Curve Fitting Quality Evaluation

The above experimental data demonstrate that the service life of polyimide enameled wire windings can be predicted through accelerated thermal aging tests. Among the most commonly used aging indicators, tan δ is susceptible to environmental factors such as humidity, making the aging patterns characterized by it difficult to apply in certain contexts. Therefore, this study did not select tan δ as the monitoring indicator for the thermal lifespan of polyimide enameled wire windings. In contrast, IC exhibits a more pronounced rate of change and higher stability. The experimental data also reveal a clear monotonic trend in IC, which shows a strong correlation with thermal lifespan degradation. Hence, this study considers IC to be a suitable indicator for evaluating the lifespan of polyimide enameled wire windings.

**Figure 17:**
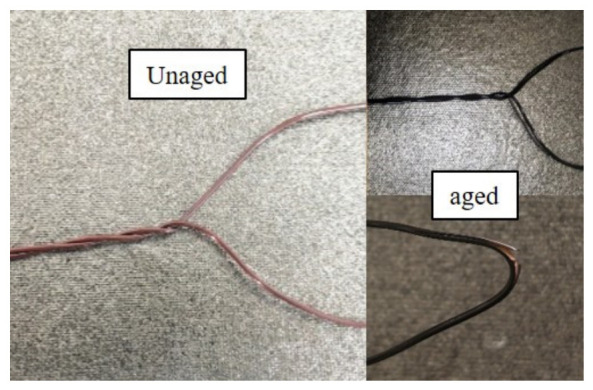
The appearance changes in polyimide enamel-insulated wires during thermal aging. (thermal aging conditions: Under atmospheric conditions, at 350 °C, after the lifespan is exhausted).

However, the two characteristics L and R, which are crucial for the electrical properties of the windings, show no clear monotonic relationship with the thermal aging of the enameled wire. From the beginning to the end of their lifespan, no significant or regular changes were observed, only minor oscillations. Throughout the thermal aging process, the amplitude of these oscillations was small and followed no fixed pattern. Therefore, it is concluded that there is no correlation between the L and R indicators of the windings and the thermal aging of the enameled wire, making them unsuitable for monitoring the thermal lifespan of polyimide enameled wire. This also demonstrates that during the insulation degradation stage of polyimide enameled wire, the aging process does not significantly affect the key electrical parameters of the windings and thus has no notable impact on the system’s electrical performance. From the perspective of the aging mechanism of the polyimide enamel layer, the insulation degradation of the enameled wire primarily results from phenomena such as the aging, decomposition, thinning, and even cracking of the organic enamel layer. This is also observable in the changes in the appearance of the enameled wire, as shown in Figure 17.

Figure 17 shows a comparison of the characteristics and color of the enameled wire before and after aging. After aging, the insulation enamel layer of the wire exhibits significant darkening and embrittlement. As long as they are not in physical contact or have suffered breakdown and conduction, the current can only flow along the original circuit of the copper wire core, so R does not change significantly. Similarly, since the number of turns and the magnetic circuit remain unchanged, L does not undergo significant changes either. Furthermore, for the aviation motors without PD (partial discharge) phenomena in this study, i.e., Type I motors, the impact of insulation degradation on the motor’s operating state is minimal before the occurrence of turn-to-turn short circuits.

Regarding the cause of the oscillations, this study rules out the possibility of excessive errors in the testing instruments or methods. It is believed that the main reason for the oscillations lies in the fact that during the accelerated thermal aging process, the coils and windings are subjected to thermal stress far exceeding their normal operating temperature. This may lead to thermal expansion and contraction of the metal wire core, generating mechanical stress, or it may release the mechanical stress accumulated during the winding process, causing slight deformation in the winding shape. Consequently, this leads to irregular oscillations in the values of L and R.

It is worth noting that in subsequent research, the characteristics after the end of the enameled wire’s lifespan can be explored. The insulation failure of the enameled wire does not mean the immediate end of the winding’s lifespan. There is still a certain health degradation process between the loss of insulation reliability of the enameled wire and the occurrence of severe short circuits in the entire winding. This process can be considered for monitoring using changes in L or R values.

Essentially, both values can be approximated as turn-to-turn capacitance, as explained earlier. Therefore, in the thermal lifespan monitoring of coil samples, the corrected aging curve obtained in this study defines the relationship between the $C_{p}$ value and the thermal lifespan.

For the evaluation of curve fitting quality, this paper employs two classic metrics: R^2^ and RMSE. The closer the R^2^ value is to 1, the higher the model accuracy, but an excessively high R^2^ value indicates a potential risk of overfitting. A smaller RMSE signifies a smaller prediction error. The evaluation results for the prior model, the corrected model, and the model fitted using only new data are presented in the table below.

Based on the comparative results in Table 2, the R^2^ of the updated model is closer to 1 and, unlike the other two, does not exhibit an excessively high value. This indicates higher accuracy and the absence of a significant overfitting risk. Meanwhile, the RMSE of the updated model is also the smallest among the three, suggesting the lowest error.

Overall, the fitting performance of the updated model is superior to that of both the prior model and the model using only the new data. This demonstrates that the proposed method successfully improves the accuracy of winding lifespan prediction, and the Bayesian nonlinear regression approach performs well with the dataset in this study.

In classical studies, the two-parameter Weibull distribution is often used to process experimental data from accelerated thermal aging tests [8,11,21]. This study innovatively adopts the method of Bayesian nonlinear regression, leveraging its capability to update prior distributions with new data. Specifically, we propose an approach that first establishes a prior distribution using twisted-pair samples and then updates this prior with coil data to obtain the posterior distribution. This method uses twisted-pair samples, which are easy to fabricate and more cost-effective, as the primary consumables. Compared to conducting aging tests entirely on aviation motor windings, this approach significantly reduces experimental costs while ensuring result accuracy. Furthermore, Bayesian nonlinear regression inherently resists the risk of overfitting, thus providing a high-quality fit.

It should be noted that this study has certain limitations. The conclusions drawn are based on experiments using twisted-pair samples and finished windings, and their accuracy has not yet been verified in actual generators. Furthermore, constrained by time and economic costs, this study was unable to establish a dataset with a larger volume of data or conduct accelerated aging tests at more temperature levels. Although the existing data are sufficient to support the conclusions of this study, a larger dataset would undoubtedly further enhance the accuracy of the findings.

## 5. Conclusions

This study focuses on polyimide enameled wire and, based on the Arrhenius law, conducts accelerated aging experiments on two classic parameters characterizing the thermal lifespan of enameled wire—IC and tan δ—as well as two parameters reflecting the fundamental electrical properties of windings, L and R. After employing B-spline interpolation for data analysis, IC (manifested as $C_{p}$ in measurements of finished windings) was ultimately selected as the most suitable parameter for characterizing the thermal lifespan of polyimide enameled wire. Using Bayesian nonlinear regression, the aging curve of $C_{p}$ versus thermal lifespan was derived, and its fitting quality was evaluated. This curve reflects the relationship between the percentage change in the winding’s $C_{p}$ and the insulation health status of the winding, providing both a qualitative methodology and quantitative reference values for monitoring the health status of polyimide enameled wire windings.

According to the experimental results, the degradation of the insulation performance of polyimide enameled wires has no significant impact on the electrical performance of the system. However, the above findings still require further validation in actual generator systems. This study has certain limitations. First, although the dataset size is sufficient to draw experimental conclusions, it is undeniable that the scale is relatively small. A larger dataset would help improve the accuracy and rigor of the research. Second, the selection of aging temperatures is relatively limited. Including more temperature levels would better verify whether the aging behavior follows the Arrhenius law. Finally, the conclusions drawn in this study still need to be validated in real aviation generator systems. Subsequent research should extend to actual aviation motor systems to verify the conclusions of this study in a complete application environment. In future studies, regarding the material selection for generator windings, insulating varnish materials and processes with stronger thermal stability and insulation performance could be explored, such as using nanocomposite polyimide materials. Furthermore, in the aforementioned analysis, thermal stress is considered the sole factor leading to insulation aging. However, under real operating conditions, other aging factors can contribute to shortened insulation life. Among these, the most relevant factors besides thermal stress are electrical stress, mechanical stress, and environmental stress. Comprehensively considering the aging conditions under multi-stress coupling and modeling the aging mechanisms at the microscale would better reflect the actual aging behavior of polyimide enameled wires.

## Figures and Tables

**Table 1:** The average thermal lifespan across three temperature conditions [22].

Temperature (°C)	310	330	350
Average Thermal Lifespan (h)	134.18	80.31	35.47

**Table 2:** Model evaluation report form.

	Prior Fitting Curve	New Data Curve	Updated Fitting Curve
$R^{2}$	0.8500	0.8196	0.8559
RMSE	0.0481	0.0528	0.0472

## Data Availability

The original contributions presented in this study are included in the article. Further inquiries can be directed to the corresponding author.

## Abbreviations

The following abbreviations are used in this manuscript:

PI	Polyimide
IC	Insulation Capacitance
tan δ	Dielectric Dissipation Factor
PDIV	Partial Discharge Inception Voltage
L	Electrical Inductance
R	Electrical Resistance
PTFE	Poly Tetra Fluoro Ethylene
$C_{p}$	Partial Discharge Inception Voltage
PD	Partial Discharge
